# Comparison of internal and external fixation of distal radius fractures: A meta-analysis of randomized controlled trials

**DOI:** 10.3109/17453674.2013.792029

**Published:** 2013-05-31

**Authors:** Xuetao Xie, Xiaoxing Xie, Hui Qin, Longxiang Shen, Changqing Zhang

**Affiliations:** Department of Orthopedic Surgery, Shanghai Sixth People’s Hospital, Shanghai Jiaotong University, Shanghai, PR China

## Abstract

**Background and purpose:**

There is no consensus on the difference in effects of internal fixation (IF) and external fixation (EF) on outcomes for the treatment of distal radius fractures. We performed a meta-analysis of randomized clinical studies.

**Methods:**

We searched the literature and included studies that compared the effects of IF and EF on the treatment of distal radius fractures. Statistically, we pooled patient data using standard meta-analytic methods. For the continuous variables, the weighted mean difference (WMD) was used. For dichotomous data, the relative risk (RR) was calculated.

**Results:**

10 studies were eligible for data extraction. The pooled data showed that compared with EF, IF led to statistically significantly better Disabilities of the Arm, Shoulder, and Hand (DASH) scores at 12 months postoperatively, recovery of forearm supination at 3 months, and restoration of volar tilt and radial inclination. IF using volar locking plates resulted in better DASH scores than EF at 3 and 6 months, but the trend diminished over time; at 12 months postoperatively, the scores were not statistically significant. Compared with EF, IF led to fewer minor surgical complications.

**Interpretation:**

For surgical treatment of distal radius fractures, IF yields better functional outcomes, forearm supination, restoration of anatomic volar tilt and radial inclination, and fewer minor complications. The patients who received IF using volar locking plates for the treatment of distal radius recovered more quickly than did patients who received EF.

Currently, surgical management for distal radius fractures mainly includes external fixation (EF) and internal fixation (IF) with plate osteosynthesis. For IF, osteosynthesis with volar locking plates has gained widespread popularity. A few randomized controlled trials (RCTs) comparing IF and EF have been published in recent years. However, the relatively small sample size (n = 30–179) in each published study made the results inconclusive.

Recently, 2 meta-analyses of RCTs have compared IF and EF for the treatment of distal radius fractures (Cui et al. 2011, Wei et al. 2012). Those analyses included RCTs comparing plate fixation with percutaneous Kirschner wire fixation (Rozental et al. 2009) or comparing open reduction and bone grafting with EF (McQueen et al. 1996). Moreover, additional studies have been reported (Grewal et al. 2011, Wilcke et al. 2011) since these earlier meta-analyses, which makes the present meta-analysis more precise.

## Methods

### Search strategy

This meta-analysis was performed following the PRISMA guidelines (Liberati et al. 2009). We performed a literature search without language restrictions on October 18, 2011 and an updated search was performed on March 12, 2012 using the phrase “distal radius fractures” with the limits “randomized controlled trial”. A second search was performed using the phrase, “Colles fracture” or “Smith fracture” with the limits “randomized controlled trial” using PubMed (1949–2011), Ovid’s MEDLINE (1946–2012), MEDLINE In Process & Other Non-Indexed Citations (updated to 12 March 2012), Web of Knowledge and EMBASE (1966–2012). Further searches using the same keywords and limitations did not provide any additional references.

We also performed a search of the Cochrane Central Register of Controlled Trials (CENTRAL). Review articles were also scanned in order to find additional eligible studies. In addition, reference lists of all primary articles and previous systematic reviews and meta-analyses were searched manually for additional publications. Duplicates were removed. Information was carefully extracted from all eligible publications independently by 2 reviewers (XX and XX); disagreements were resolved by discussion between them. If a consensus could not be reached, a third investigator (CZ) adjudicated. The search results were then screened on the basis of the following inclusion criteria: (1) only randomized controlled studies on patients with distal radius fractures, (2) studies comparing IF with EF, and (3) follow-up of patients for at least 12 months. Exclusion criteria included non-randomized trials, duration of less than 12 months, and studies including children. The Jadad scale was used to assess the quality of the RCTs included, where a score of < 3 indicated low quality (Jadad et al. 1996).

### Statistics

We measured Disabilities of the Arm, Shoulder, and Hand (DASH) score, which was selected as the primary outcome, and the outcomes of forearm range of motion (ROM), grip strength, and radiographic and complication parameters. For studies that did not report ROM or strength outcomes in terms of percentages of the uninjured wrists, we tried to contact the authors to obtain this information. Where authors provided ranges instead of standard deviations of means (Xu et al. 2009), the standard deviations were approximated by the rule-of-thumb range divided by 4. For ROM or strength outcomes, impairment and percentages were calculated according to Wei et al. (2012). Radiographic parameters extracted from eligible studies were pooled by calculating absolute values or calculated from normative values for the uninjured wrist. The following normative values were used: 22° of radial inclination, 10° of volar tilt, and 11 mm of radial height.

Complications were graded as minor or major, as described by Rozental et al. (2009). Minor complications included transient extensor tendon irritation, superficial infections, and finger stiffness. Major complications included loss of reduction, malunion, and nonunion as well as deep infection, neuropathy, and tendon rupture.

We performed independent analyses for volar locking plates versus EF instead of subgroup analysis, in order to avoid result-related choices. We did not perform independent analysis for non-volar locking plates since they contained different types of plates. For the multi-arm trial (Wei et al. 2009), the combined mean and SD data were obtained following the guidance of the Cochrane Handbook for Systematic Reviews of Interventions (Higgins and Green 2011).

For the meta-analysis of continuous variables, the weighted mean difference (WMD) with 95% confidence interval (CI) was used. For dichotomous variables, the relative treatment effect was expressed as relative risk (RR) with CI. Statistical heterogeneity was investigated using the chi-square test and quantified using the I^2^ statistic. We anticipated the presence of clinical heterogeneity, based on the findings that the effects of surgery seemed to vary depending on the type of fracture, the extent of metaphyseal comminution, the quality of the bone, and the medical condition of the patient (Cherubino et al. 2010). Because the test for heterogeneity had low statistical power, we assumed the presence of heterogeneity a priori, and used the random-effects model in all pooled analyses. A sensitivity analysis was conducted by detecting the effect of individual studies on the pooled effect size. Funnel plots and Egger’s tests were used to assess possible publication bias. Any p-value less than 0.05 was considered to be statistically significant. Analyses were performed using the Stata/SE 10.0 program for Windows (Stata Corporation, College Station, TX). The user-written commands for meta-analysis (Harris et al. 2008) were downloaded from within Stata.

## Results

### Selected studies and characteristics

**Figure 1.**
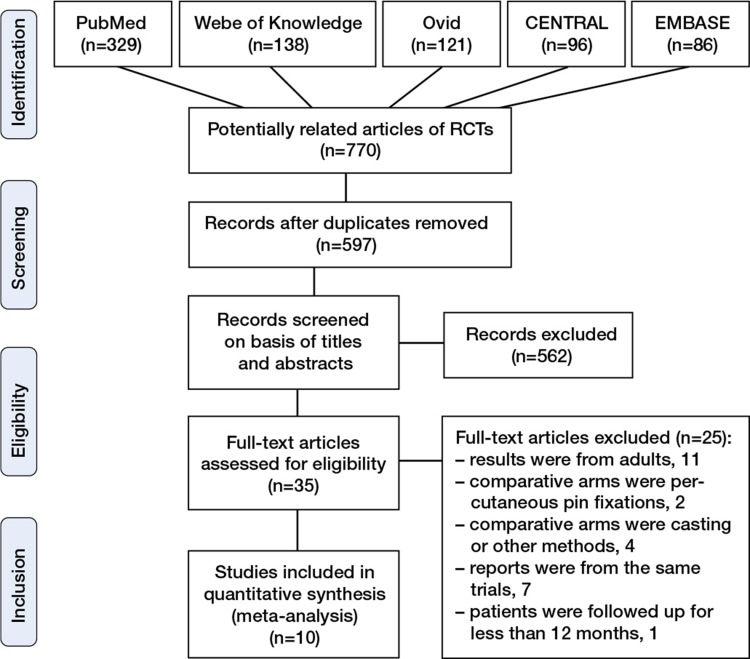
Flow chart of the meta-analysis.

770 potentially relevant citations were identified and screened, of which only 10 published RCTs met the inclusion criteria and were selected for this meta-analysis (Kapoor et al. 2000, Grewal et al. 2005, Kreder et al. 2005, Egol et al. 2008, Leung et al. 2008, Abramo, et al. 2009, Wei et al. 2009, Xu et al. 2009, Grewal et al. 2011, Wilcke et al. 2011) (Figure 1 and Table 1). One RCT was excluded from our analysis because of a duration of only 6 months after fixation (Jeudy et al. 2012).

**Table 1.** Basal characteristics of clinical trials included in the analysis

Author Year	Age	No. of fract. (EF/IF)	Femal (EF/IF)	Classification of fractures	IF	EF	Follow-up (months)	Outcomes measured	Jadad score
Grewal et al. 2011	18–75	26 /27	18/20	AO type A, C1, C2, C3	Volar locking plates and dorsal Pi plates	Bridging fixators + suppl. K-wires	12	PRWE and DASH scores, grip strength, ROM, radiographic results and complications.	3
Wilcke et al. 2011	20–70	30 /33	23/25	AO type A, C1	Volar locking plates	Bridging fixators ± suppl. K-wires	12	PRWE and DASH scores, grip strength, ROM, radiographic results and complications.	3
Wei et al. 2009	>18	22/12	16/8	AO type A3, C1, C2, C3	Locked radial column plates and volar locking plates	Bridging fixators + suppl. K-wires	12	DASH score, grip and lateral pinch strength, ROM, pain scores, radiographic results and complications.	4
Abramo et al. 2009	20–65	24/26	17/19	AO type A1–3, C1–3	The TriMed system	Bridging fixators + suppl. K-wires	12	DASH score, grip strength, ROM, pain scores, radiographic results and complications.	3
Xu et al. 2009	21–56	14/16	5/7	AO type C	A variety of non-locking plates.	Bridging fixators ± suppl. K-wires	24	The Green and O’Brien scoring, grip strength, ROM, radiographic results and domplications.	2
Egol et al. 2008	18–87	44/44	22/25	AO type A, B, C	Volar locking plates	Bridging fixators ± suppl. K-wires	12	DASH score, grip strength, ROM, radiographic results and complications.	3
Leung et al. 2008	17–60	74/70	—	AO type C1, C2, C3	Nonlocking T-plates, combined volar and dorsal plate fixation**^a^**	Bridging fixators ± suppl. K-wires	24	Gartland and Werley point system, Green and O’Brien clinical score, arthritis grade, daily activities, social activities, interference of normal work and complications.	3
Grewal et al. 2005	<70	33/29	12/17	AO type C	Locked dorsal Pi plate	Bridging fixators + suppl. K-wires	24	DASH scores, SF36 scores, grip and pinch strength, ROM, radiographic results, surgical outcomes and complications.	2
Kreder et al. 2005	16–75	88/91	38/32	AO type B, C	Small or mini-fragment plate	Bridging fixators ± suppl. K-wires	24	MFA function test, SF36 scores, Jebsen score, grip strength and pinch strength, three-jaw chuck and pad-to-pad pinch strength, ROM, radiographic results and complications.	3
Kapoor et al. 2000	—	28/29	8/10	Frykman’s type III, IV, VII, VIII	Small T plates	Bridging fixators	48	Grip strength, radiographic results, Sarmiento functional score and complications.	1

**^a ^**The latter was done for more comminuted fractures PRWE: Patient Rated Wrist Evaluation. DASH: Disabilities of the Arm, Shoulder, and Hand. ROM: Range of motion.

The level of evidence for each article was graded from scores 1 to 4 according to the Jadad quality score (Jadad et al. 1996). 715 patients with 772 fractures were included in this analysis. 1 study (Xu et al. 2009) was part of a multicenter trial (Leung et al. 2008), and both studies were included in the analysis since they reported different types of data. Allocation concealment was adequately reported in 4 trials and was unclear in the remaining trials. Because of the obvious nature of the intervention, no trials were double-blind.

### Comparison of the effects of internal fixation and external fixation on functional outcome

**Figure 2.**
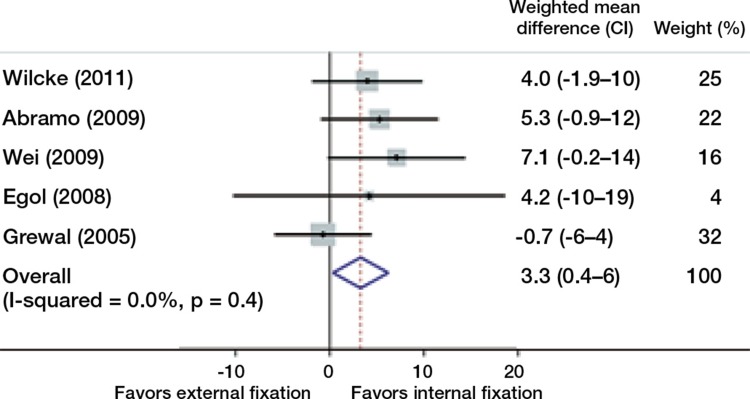
Comparison of the effects of internal and external fixation on DASH score at 12 months after surgery. (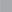) The weighting given to the trial in the overall pooled estimate, taking into account the number of participants and the amount of between-study variation (heterogeneity). (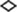) The combined effect size.

The pooled results of our primary outcome measure, the DASH scores, presented a significant difference favoring IF over EF at 12 months postoperatively (Table 2, see Supplementary data, and Figure 2), but not at 3 and 6 months postoperatively.

An independent analysis of randomized studies comparing IF using volar locking plates with EF showed that at 3 and 6 months, the volar plate group had better DASH scores, but the difference diminished over time; at 12 months, the scores were not statistically significantly different and showed a trend in favor of EF (Table 3).

**Table 3.** Comparison of internal fixation (IF) using volar locking plates and external fixation (EF) regarding the outcomes of DASH, grip strength, and 12-month radiological results

			Fractures					
Time	Time or parameters	Studies	EF	IF	Weighted mean difference	95% CI	p-value	Favored
DASH	3 months	4	112	102	14.3	7 to 22	< 0.001	IF
	6 months	4	113	102	6.2	3 to 9	< 0.001	IF
	12 months	4	113	102	6.1	-0.7 to 13	0.08	
Grip strength	3 months	3	90	84	-14	-25 to -3	0.01	IF
	6 months	3	90	84	3.1	-18 to 24	0.8	
	12 months	3	90	84	-1.8	-16 to 12	0.8	
12-month	Volar tilt	2	60	63	-4.7	-7 to -2	< 0.001	IF
radiological	Radial length	2	60	63	1.0	0.1 to 2	0.03	EF
results	Radial inclination	3	90	96	1.0	-1 to 4	0.4	
	Ulnar variance	3	90	96	-0.1	-2 to 2	1	

95% CI, 95% confidence interval; DASH: Disabilities of the Arm, Shoulder, and Hand.

### Comparison of the effects of internal fixation and external fixation on grip strength and ROM of wrist and forearm

Grip strength analysis across 6 to 8 studies at 3, 6, and 12 months of follow-up showed that there were no statistically significant differences between IF and EF (Table 2, see Supplementary data) and that use of a volar locking plate predictably leads to better grip strength in the first 3 months after fixation. However, at 6 months and 1 year, the outcomes of both techniques evaluated in this study were similar (Table 3). The study by Kapoor et al. (2000) provided mean grip strength values without standard deviations, and could not therefore be included.

Pooling of wrist and forearm ROM data was possible across 8 of the 10 studies. Kapoor et al. (2000) provided ROM data without standard deviations, and these data were not included. A statistically significant difference in supination was found in favor of plate fixation at 3 months postoperatively (WMD = –11, CI: –16 to –7; p < 0.001) (Figure 3, see Supplementary data). No other ROM variables revealed any significant differences in treatment effect at 3, 6, or 12 months after fixation (Table 4, see Supplementary data). For comparison of IF with volar locking plates and EF, the pooled data across 3 to 4 studies showed that the supination, extension was superior in the volar locking plates group 3 months after fixation, and there was better flexion 6 months after fixation. However, at 12 months, the ROM data were similar for these 2 treatment methods (Table 5, see Supplementary data).

### Comparison of the effects of internal fixation and external fixation on radiographic parameters

Meta-analysis showed that volar tilt and radial inclination were superior for IF than for EF 12 months postoperatively (Table 2, see Supplementary data). The other parameters—radial length and ulnar variance—showed no statistically significant differences between the groups. For comparison of volar locking plates and external fixators, volar tilt was superior with volar locking plates and radial length was superior with external fixators 12 months postoperatively. The other parameters—radial inclination and ulnar variance—were similar between the 2 groups (Table 3).

### Comparison of the effects of internal fixation and external fixation on final complications

Because the study by Xu et al. (2009) was part of the study by Leung et al. (2008), and both papers reported data on complications, only Leung’s data were included. The 9 eligible studies had reports of 728 fractures with information on total surgical complications (Figure 4, see Supplementary data). The pooled results showed that the complications with IF were not statistically significantly different to those with EF (RR = 1.2, CI: 0.87–1.7; p = 0.3). Further analyses indicated that minor complications in the IF group were less than in the EF group (RR = 3.6, CI: 2.0–6.7; p < 0.001) (Figure 5, see Supplementary data), while no significant difference in major complications was detected (RR = 0.79, CI: 0.55–1.2; p = 0.2). For comparison of IF with volar locking plates and EF, the 3 eligible research studies covering 174 fractures demonstrated that the total number of complications with volar locking plates and with external fixators was similar (RR = 1.29, CI: 0.64–2.6; p = 0.5) (Figure 4, see Supplementary data). Further analyses showed similar results in both minor and major complications (RR = 2.7, CI: 0.82–9; p = 0.06 and RR = 0.86, CI: 0.38–2.0; p = 0.7).

### Sensitivity analysis and publication bias analysis

For the complications data that most studies included in their analysis, we evaluated the influence of any individual study on the overall RR. No individual study affected the overall RR predominantly, since omission of any single study did not make a large difference. Moreover, Begg’s funnel plot and Egger’s test were performed to evaluate the publication bias in the literature. The shapes of the funnel plots did not reveal any evidence of obvious asymmetry (Figure 6, see Supplementary data), and Egger’s test suggested the absence of any publication bias (p = 0.3).

## Discussion

Our results indicate that IF leads to statistically significantly better DASH scores than EF 12 months after surgery. For comparison of volar locking plates and EF, the DASH was improved at the early stage after fixation. Possible explanations are that IF is more reliable and reproducible in achieving an anatomic reduction than EF, which is supported by the radiographic results in our analysis. Furthermore, the fixed-angle nature of volar locking plates achieves adequate stability for unstable distal radius fractures (Kamei et al. 2010) and allows early wrist mobilization, leading to improved strength. As shown in the grip strength outcomes in our analysis, the volar locking plates group showed significantly better grip strength than EF at 3 months after fixation. Since better functional results can be expected in the early postoperative period with volar locking plates, this form of fixation should be considered for patients requiring a faster return to function after injury.

IF led to better recovery of supination at 3 months after surgery. Further independent analysis indicated that volar locking plates restored better supination and extension at 3 months and flexion at 6 months postoperatively. To our knowledge, no previous meta-analysis has analyzed data from RCTs comparing IF with volar locking plates and EF.

Malunion of distal radius fractures has been shown to lead to poor functional outcome (Grewal and MacDermid 2007, Brogren et al. 2011). Our analysis shows that IF restored volar tilt and radial inclination significantly better than EF, with independent analysis concerning IF using volar locking plates supporting this conclusion. On the other hand, compared to IF with volar locking plates, EF remains a good option for restoration and maintenance of radial length after fractures of the distal radius.

Our pooled data showed that both IF and IF using volar locking plates were not advantageous regarding total or major complications, while IF only led to lower minor complications after surgery. These results are different from those in the previous meta-analysis (Cui et al. 2011), which found a statistically significantly reduced risk of total surgical complications with IF in comparison with EF. With only eligible studies included in the analysis and including the data from multi-arm study, and after correction of improper data, sensitivity analysis indicated that our result was stable—and funnel plot and Egger’s test indicated that there was no publication bias. We believe that the results of our meta-analysis are more precise than those recently published.

Certain limitations in the present meta-analysis should be considered. First, the analysis was based on published data only, and no unpublished data were included. Also, heterogeneity in patient age should be expected because it was impossible to match the cohorts completely for the analysis. In patients who were 65 years of age or older, the risk of adverse outcomes in extra-articular distal radius fractures was less (Grewal et al. 2007), and anatomic reduction did not give any improvement in terms of ROM or the ability to perform activities of daily living (Arora et al. 2011). However, there were no studies included in our meta-analysis that compared the effects of age on the outcomes of IF and EF, so we were unable to do a pooled analysis. On the other hand, our meta-analysis did not reveal differences in fracture type-specific effects between IF and EF on the treatment of distal radius fractures due to the limited number of trials.

Finally, the performance of activities of daily living and vocational function and economic impact parameters such as time off work, ability to return to previous occupation, and duration and cost of postoperative rehabilitation were not measured in the majority of the studies included, so we are unable to perform a pooled analysis involving these considerations. These factors limit our ability to compare the effects of IF and EF on distal radius fracture treatment.

We conclude that in managing distal radius fractures, IF was superior with EF. The patients who received IF with volar locking plates for the treatment of distal radius fractures recovered more quickly than did patients who received EF. In further research, one could compare the effects of IF with volar locking plates and those of EF in multicenter trials on different types of fractures—trials containing both young and old patients, with economic analysis performed in parallel to attain robust evidence.

## Supplementary data

Tables 2, 4, and 5, and Figures 3–6 are available at our website (www.actaorthop.org), identification number 5546.
