# Supplementary material for: Comparison of internal and external fixation of distal radius fractures: A meta-analysis of randomized controlled trials
Source: Acta Orthop. 2013 May 31;84(3):286–91. doi: 10.3109/17453674.2013.792029 (PMC3715819; doi:10.3109/17453674.2013.792029)
Supplement: Supplementary file 1 [file ORT-84-286-s5546.pdf]

Supplementary article data

Comparison of internal and external fixation of distal radius fractures

A meta-analysis of randomized controlled trials

Xuetao Xie, Xiaoxing Xie, Hui Qin, Longxiang Shen, and Changqing Zhang

Department of Orthopedic Surgery, Shanghai Sixth People's Hospital, Shanghai Jiaotong University, Shanghai, PR China
Correspondence: shenlongxiang@yahoo.cn
Submitted 12-03-29. Accepted 12-11-11

Table 2. Comparison of internal fixation (IF) and external fixation (EF) regarding the outcomes of DASH, grip strength, and 12-month radiological results

Time	Time or parameters	Studies	Fractures EF	Fractures IF	Weighted mean difference	95% CI	p-value	Favored
DASH	3 months	5	147	151	7.3	-0.2 to 15	0.06	
	6 months	4	123	125	1.5	-6 to 9	0.7	
	12 months	5	147	151	3.3	0.4 to 6	0.03	IF
Grip strength	3 months	6	169	176	-6.5	-15 to 2	0.1	
	6 months	7	183	192	3.2	-7 to 13	0.5	
	12 months	8	271	283	1.5	-6 to 9	0.7	
12-month radiological results	Volar tilt	5	131	134	-2.0	-4 to -0.5	0.01	IF
	Radial length	4	107	108	-0.8	-2 to 0.5	0.2	
	Radial inclination	7	185	193	-0.8	-2 to -0.1	0.04	IF
	Ulnar variance	7	185	189	0.5	-0.5 to 1.5	0.3	

95% CI, 95% confidence interval;
DASH: Disabilities of the Arm, Shoulder, and Hand.

Table 4. Comparison of internal fixation (IF) and external fixation (EF) regarding range of motion (ROM) results

Time	ROM results	Studies	Fractures EF	Fractures IF	Weighted mean difference	95% CI	p-value	Favored
3 months	Supination	6	171	177	-11.3	-16 to -7	< 0.001	IF
	Pronation	6	171	177	-5	-11 to 2	0.1	
	Extension	6	171	177	-6.6	-15 to 2	0.1	
	Flexion	6	171	177	-3.1	-8 to 2	0.3	
	Radial deviation	6	171	177	-3.5	-21 to 14	0.7	
	Ulnar deviation	6	171	177	-2.3	-9 to 4	0.5	
6 months	Supination	8	273	284	-3.3	-8 to 2	0.2	
	Pronation	8	273	284	-3.3	-15 to 7	0.5	
	Extension	8	273	284	-2.7	-14 to 8	0.6	
	Flexion	8	273	284	-2.3	-6 to 2	0.3	
	Radial deviation	8	273	284	3.5	-6 to 13	0.5	
	Ulnar deviation	8	273	284	2.8	-1 to 6	0.1	
12 months	Supination	8	273	284	-2.1	-6 to 2	0.3	
	Pronation	8	273	284	-2.3	-5 to 0.7	0.1	
	Extension	8	273	284	0.4	-4 to 5	0.9	
	Flexion	8	273	284	0.8	-2 to 4	0.6	
	Radial deviation	8	273	284	-0.3	-7 to 6	0.9	
	Ulnar deviation	8	273	284	0.6	-5 to 6	0.9	

95% CI, 95% confidence interval

Open Access - This article is distributed under the terms of the Creative Commons Attribution Noncommercial License which permits any noncommercial use, distribution, and reproduction in any medium, provided the source is credited.
DOI 10.3109/17453674.2013.792029

Table 5. Comparison of internal fixation (IF) using volar locking plates and external fixation (EF) regarding range of motion (ROM) results

Time	ROM results	Studies	Fractures EF	Fractures IF	Weighted mean difference	95% CI	p-value	Favored
3 months	Supination	3	90	96	-13.2	-19 to -7	< 0.001	IF
	Pronation	3	90	96	-5.3	-15 to 5	0.3	
	Extension	3	90	96	-14.6	-24 to -6	< 0.001	IF
	Flexion	3	90	96	-4.3	-13 to 5	0.3	
	Radial deviation	3	90	96	-10.3	-40 to 20	0.5	
	Ulnar deviation	3	90	96	-1.9	-10 to 6	0.6	
6 months	Supination	3	90	96	-6.1	-13 to 1	0.1	
	Pronation	3	90	96	-17.9	-47 to 12	0.2	
	Extension	4	123	125	-7.8	-23 to 7	0.3	
	Flexion	3	90	96	-8.4	-14 to -3	< 0.001	IF
	Radial deviation	3	90	96	3.4	-16 to 22	0.7	
	Ulnar deviation	3	90	96	4.4	-2 to 11	0.2	
12 months	Supination	3	90	96	0.2	-7 to 7	1	
	Pronation	3	90	96	-3.5	-8 to 0.6	0.1	
	Extension	3	90	96	-4.9	-14 to 5	0.3	
	Flexion	3	90	96	-0.5	-5 to 5	0.9	
	Radial deviation	3	90	96	1.4	-5 to 7	0.6	
	Ulnar deviation	3	90	96	-5.2	-15 to 4	0.3	

95% CI, 95% confidence interval

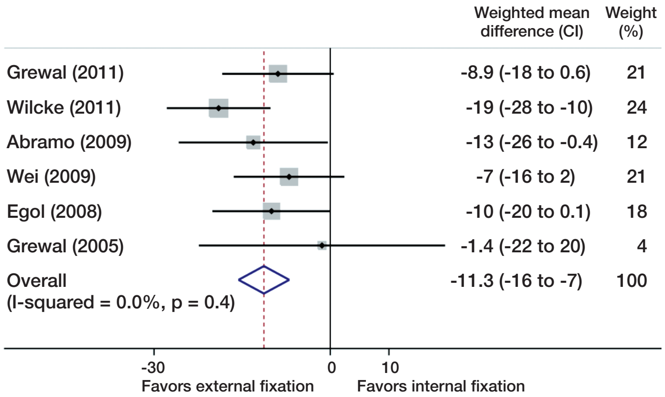

Figure 3. Comparison of the effects of internal and external fixation on supination at 3 months after surgery. (■) The weighting given to the trial in the overall pooled estimate, taking into account the number of participants and the amount of between-study variation (heterogeneity). (◇) The combined effect size.

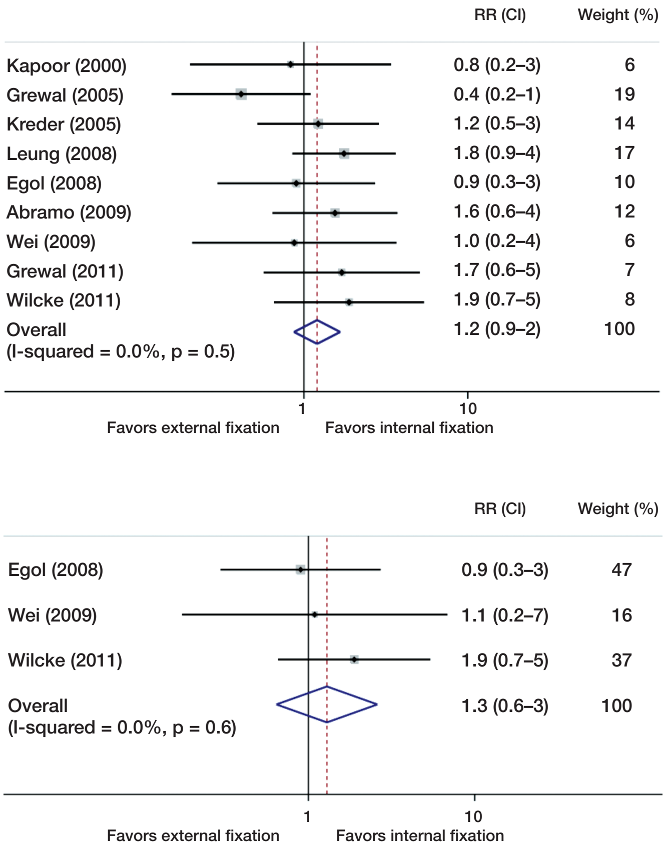

Figure 4. Comparison of the final complications of internal and external fixation. Upper graph internal fixation versus external fixation. Bottom graph internal fixation using volar locking plates versus external fixation. (■) The weighting given to the trial in the overall pooled estimate, taking into account the number of participants and the amount of between-study variation (heterogeneity). (◇) The combined effect size.

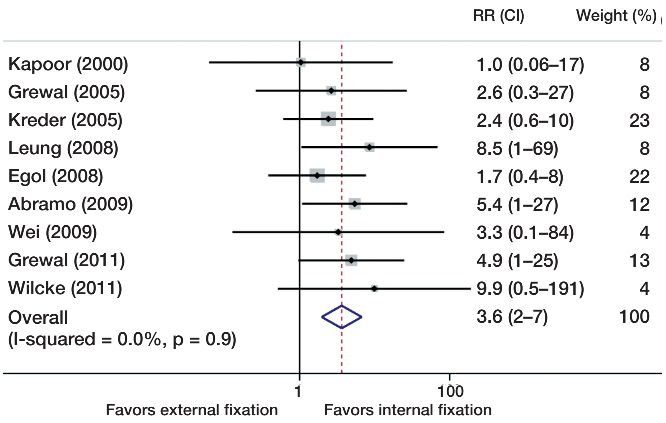

Figure 5. Comparison of the minor complications of internal and external fixation. (■) The weighting given to the trial in the overall pooled estimate, taking into account the number of participants and the amount of between-study variation (heterogeneity). (◇) The combined effect size.

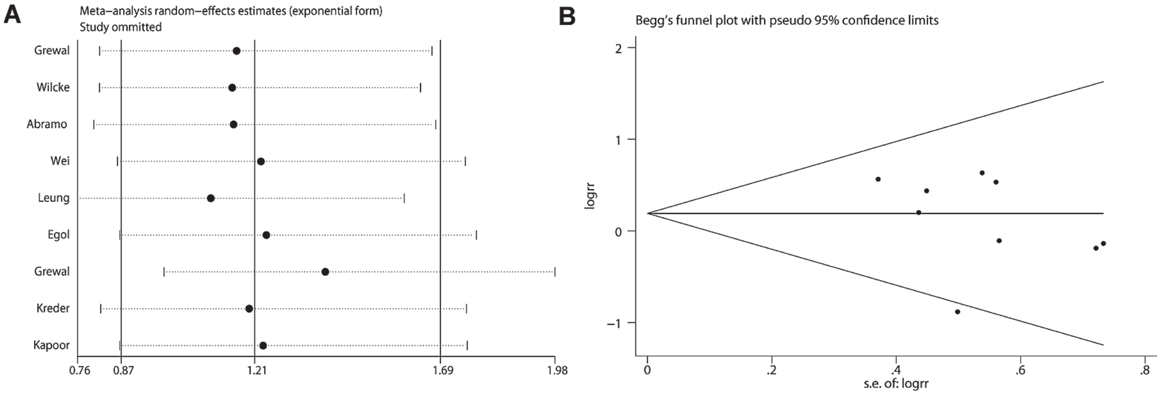

Figure 6. Sensitivity analysis and publication bias analysis for the analysis. A. The influence of individual studies on the summary RR. The vertical axis indicates the overall RR and the 2 vertical axes indicate its 95% CI. Every circle indicates the pooled RR when the study is omitted in this meta-analysis. The 2 ends of every broken line represent the respective 95% CI. B: Begg's funnel plot of studies included in the meta-analysis. The vertical axis represents log [RR] and the horizontal axis means the standard error of log [RR]. Horizontal line and sloping lines in the funnel plot represent effects summary RR and expected 95% CI for a given standard error, respectively. Each circle represents an independent study.
